# A patient-specific study of type-B aortic dissection: evaluation of true-false lumen blood exchange

**DOI:** 10.1186/1475-925X-12-65

**Published:** 2013-07-06

**Authors:** Duanduan Chen, Matthias Müller-Eschner, Hendrik von Tengg-Kobligk, David Barber, Dittmar Böckler, Rod Hose, Yiannis Ventikos

**Affiliations:** 1 Department of Biomedical Engineering, School of Life Science, Beijing Institute of Technology, Beijing, China; 2 Department of Diagnostic and Interventional Radiology, University Hospital Heidelberg, Heidelberg, Germany; 3 Radiology, German Cancer Research Center (dkfz), Heidelberg, Germany; 4 Institute for Diagnostic, Interventional and Pediatric Radiology (DIPR), Inselspital, University Hospital, Bern, Switzerland; 5 Department of Cardiovascular Science, Medical Physics Group, University of Sheffield, Sheffield, UK; 6 Department of Vascular Surgery, University of Heidelberg, Heidelberg, Germany; 7 Department of Mechanical Engineering, University College London, London, UK

**Keywords:** Aortic dissection, Computational fluid dynamics, Patient-specific model, Hemodynamics

## Abstract

**Background:**

Aortic dissection is a severe pathological condition in which blood penetrates between layers of the aortic wall and creates a duplicate channel – the false lumen. This considerable change on the aortic morphology alters hemodynamic features dramatically and, in the case of rupture, induces markedly high rates of morbidity and mortality.

**Methods:**

In this study, we establish a patient-specific computational model and simulate the pulsatile blood flow within the dissected aorta. The k-ω SST turbulence model is employed to represent the flow and finite volume method is applied for numerical solutions. Our emphasis is on flow exchange between true and false lumen during the cardiac cycle and on quantifying the flow across specific passages. Loading distributions including pressure and wall shear stress have also been investigated and results of direct simulations are compared with solutions employing appropriate turbulence models.

**Results:**

Our results indicate that (i) high velocities occur at the periphery of the entries; (ii) for the case studied, approximately 40% of the blood flow passes the false lumen during a heartbeat cycle; (iii) higher pressures are found at the outer wall of the dissection, which may induce further dilation of the pseudo-lumen; (iv) highest wall shear stresses occur around the entries, perhaps indicating the vulnerability of this region to further splitting; and (v) laminar simulations with adequately fine mesh resolutions, especially refined near the walls, can capture similar flow patterns to the (coarser mesh) turbulent results, although the absolute magnitudes computed are in general smaller.

**Conclusions:**

The patient-specific model of aortic dissection provides detailed flow information of blood transport within the true and false lumen and quantifies the loading distributions over the aorta and dissection walls. This contributes to evaluating potential thrombotic behavior in the false lumen and is pivotal in guiding endovascular intervention. Moreover, as a computational study, mesh requirements to successfully evaluate the hemodynamic parameters have been proposed.

## Background

Aortic dissection (AD) can present a challenging clinical emergency of the human aortic system [1]. The incidence of AD normally results from the combined effects of an initiating event and a structural weakness of the arterial wall [2]. It is often associated with injury, infection, congenital weakness of the aorta, collagen disorders such as Marfan’s syndrome, abdominal aortic aneurysm, etc. [3-5]. The establishment of AD is commonly initiated by the dilatation of the aorta or high blood pressures which tear the intima, allowing a surge of blood to flow into the aortic wall; the pulsatile pressure of the circulation then drives the blood and separates the layers of the aortic wall, resulting in the formation of a false lumen within the separated area of the original wall of the aorta. Although the disease is uncommon, the outcome of AD is frequently fatal. Risks include atherosclerosis and hypertension, and death is usually caused by acute aortic regurgitation, major branch vessel obstruction, or aortic rupture [6].

Aortic dissection is suspected in patients with anterior chest and back pains that progress downward. Clinical diagnosis is commonly confirmed by Computed Tomography (CT) [7,8]. Magnetic resonance imaging (MRI) allows making the diagnosis as well but is less suited in the acute setting, transoesophageal echocardiography (TEE) is used to confirm diagnosis intraoperatively. Medical management of AD involves antihypertensive treatment at the initial stage, surgical treatment to excise and replace the aortic segment that contains the origin of the dissection, and treatment with endovascular stent placement. These treatments have already made considerable progress in clinical management of AD; however, the mortality rate of this disease remains high, e.g. 70% during operations [6]. Better understanding of the hemodynamics of the dissected aorta is still needed.

In recent years, computational hemodynamics has been increasingly used in analyzing the diseases of the cardiovascular system, in assistance of clinical studies [9-14], but only a few studies have focused on aortic dissection. In the studies of AD, some of the computational models are based on artificially designed geometries [15,16]; however, patient-specific models (based on medical imaging) can provide more accurate and convincing information [17]. Recent studies by Cheng and colleagues [18] and Tse et al. [19,20] have provided quantitative assessments of the hemodynamics in the AD; by presenting the velocity and loading distributions within the dissected aorta, they furthered our knowledge regarding the pathogenesis of this disease. Moreover, other computational studies by Cheng et al. [21] and Karmonik et al. [22-24] focused the AD treated by endovascular stentgraft, revealing the fluidic environment of the dissected aorta pre- and post-interventionally. The abovementioned studies improved our understandings regarding this disease and the relevant treatments; however, detailed information regarding the flow communication between the true and false lumina has not been reported. Since the inter-luminal flow exchange is highly related to the enlargement of the false lumen, and the success for one of the most common interventions for this disease, endovascular stentgraft implantation, depends strongly on accurate knowledge of the characteristics of this exchange, we believe quantitative analyses concerning the flow communication between the two channels of AD are still needed.

In this study, we establish a computational model of a dissected aorta by reconstructing the surface from a CT dataset. The computed region starts at the ascending aorta and ends above the diaphragm. By solving the three-dimensional unsteady conservation equations for mass and momentum, we provide detailed information about hemodynamics of the aortic dissection system. Velocity, pressure and wall shear stress distributions are reported and salient hemodynamic features are identified, however our main focus is the identification of blood exchange and crossflow between the true and the false lumen. This exchange occurs through a series of (clearly identified for this case) openings and the proportions of the fluid entering each entry have been investigated. Finally, because our goal is the clinical applicability of such a technique, we investigated the behavior of finely resolved laminar computations against the solutions in the turbulent flow regime and have reached conclusions regarding mesh requirements for successful evaluation of hemodynamic parameters of interest.

## Methods

### Acquisition and reconstruction

**Figure 1:**
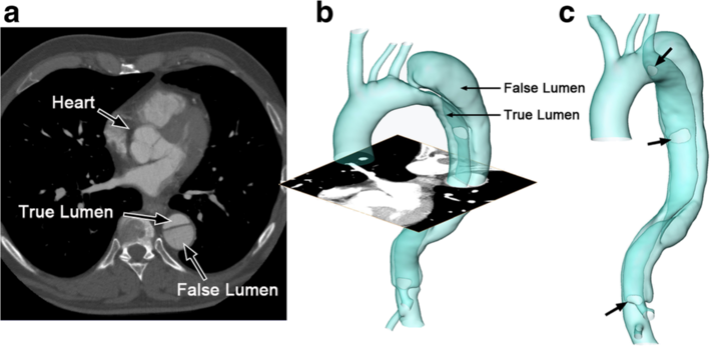
**The reconstructed surface of the aortic dissection. (a)** is one axial slice of the CT scan; **(b)** is the reconstructed surface of the aortic dissection; **(c)** shows the positions of the entries along the flap (indicated by arrows).

Formal consent from the examined patient and approval of the local ethic committee was obtained prior to the examination (Ethikkommission, Medizinische Fakultät Heidelberg: S-101/2009). A patient with Stanford type-B aortic dissection has been studied under ECG-triggered CT angiography using a 16-row multislice scanner (Aquilion 16®; Toshiba Medical Systems, Otawara, Japan) during inspiratory breath-hold with the following parameters: collimation 16mm × 1mm, tube rotation time 0.4 s, pitch 0.2, tube voltage 120 kV, tube current 300 mA, field of view 320 mm. For enhanced vessel contrast, 130 ml of a non-ionic iodinated contrast medium with an iodine content of 300 mg/ml at an injection rate of 4 ml/s (Iomeprol, Iomeron 300®; Bracco International, Milan, Italy) was used. Figure 1a displays one axial slice of the CT dataset, a cross-section in the middle of the thoracic aortic dissection. The images in this dataset have a resolution of 0.625 × 0.625mm and the inter-slice distance is 0.8 mm. 773 axial images, covering the entire dissected aorta, were analyzed.

The segmentation and surface reconstruction of the AD were accomplished by a semi-automatic, registration-based, segmentation tool – the Sheffield Image Registration Toolkit (ShIRT) [25]. ShIRT has been previously used to perform a volumetric registration of a pseudo-image, which is produced from an idealized template mesh with high quality, to the medical images, hopefully preserving the optimal characteristics of the idealized mesh under the transformation to the patient-specific case [26]. This method cannot easily be applied to the aortic dissections, since their anatomical variability is such that it is not possible to define an appropriate template that is sufficiently representative of the range of cases anticipated. For the current work a novel Region-Growing by Registration method was developed, using the same core registration algorithm, which creeps along the vessel segmenting it as it goes. The region-growing process starts by identifying a cross-section within the interested region, *i.e.* aorta or dissection, and performs multiple registration operations until the entire vessel/dissection is segmented. This sacrifices the possibility to morph a template mesh of high quality, but is much more general in terms of the geometries that it can segment. Detailed views of the reconstructed surface of the vasculature are shown in Figure 1b and c. There is a primary entry close to the aortic arch and two re-entries on the flap along the descending aorta, where blood exchange occurs between the true and false lumen. A narrow channel is established at the bottom of the dissection, which is perhaps a celiac trunk supplied by the false lumen.

The reconstructed geometry is meshed in ICEM (Ansys Inc., USA) with tetrahedral elements in the core region and prismatic cells in the boundary layer near the wall. 10 layers of prismatic cells define the near-wall region. The basic computational model, used in the turbulent computations, has 1,953,566 cells in total, however several different discretizations were used (and reported here) for grid independence verification and for the direct laminar computations.

### Numerical model

**Figure 2:**
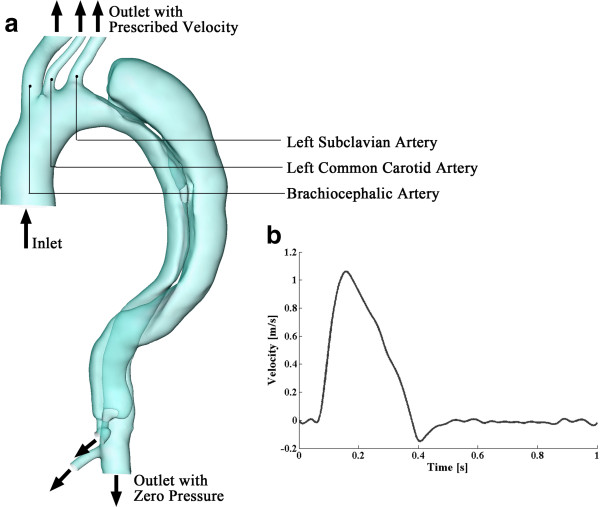
**Boundary settings of the model. (a)** describes the boundary conditions at each inlet and outlets; **(b)** displays the cross-sectional velocity profile of the inlet at the ascending aorta. Data extracted from [27].

A flat velocity profile was assigned at the inlet of the ascending aorta. The time-variant inlet velocity was derived from experimental measurements, in which the pulse wave velocity of the ascending aorta was estimated (via MRI) in 13 volunteers by a transit-time technique [27]. The representative velocity waveform is shown in Figure 2b. It presents a fundamental frequency of 1Hz with a systolic peak velocity of 1.06m/s. In the human aortic system, approximately 5% of the flow volume is diverted to each of the three aortic arch branches [28,29]. Taking into account of the cross-sectional areas of the inlet and the three aortic arch branches, the instantaneous velocities of the blood that come out from each of the three arteries can be approximated. These outflows were assigned as velocity boundary conditions for the aortic arch branches. It is assumed that the temporal waveform at each of these branches is a scaled version of that at the inlet; the peak velocities are 0.30286, 1.8239, and 0.71587 m/s for the brachiocephalic artery, left common carotid artery and left subclavian artery, respectively. Zero-pressure boundary conditions were assigned at the three other outlets of the model, as shown in Figure 2a. Since the distensibility of vessel walls is reported to be seriously reduced in aortic dissection cases [30], in this model, we assume the vessel walls are rigid with no-slip boundary conditions. Moreover, inspection of cine-sequences reveal that although for some dissections the flap oscillates substantially, for this case the oscillation is negligible and thus we assume that the flap is fixed. We believe that these assumptions (boundary conditions and rigid walls), although not exact, are particularly meaningful in a clinical setting where guidance is sought regarding hemodynamic features without the option of prolonged acquisition and computation times.

Regarding the flow, since we are investigating the flow within large arteries, the blood was treated as Newtonian and incompressible [31] with a density of 1044 kg/m^3^ and a dynamic viscosity of 0.00365 kg/m·s. Considering the inlet diameter of approximately 33.6 mm and the peak velocity of 1.06 m/s, the systolic peak Reynolds number can be calculated as 10,187, while the time-averaged Reynolds number is 1,553, indicating the blood flow within the aorta possibly at a transitional state, with possible turbulent patches and laminar regions. The k-ω SST turbulence model [32,33] is therefore employed to represent the flow. This turbulence model combines favorable features of both k-ϵ [34] and k-ω [35] models. It applies the k-ω formulation in the inner part of the boundary layer, ensuring the model directly solves the region near the wall through the viscous sub-layer, and switches to a standard k-ϵ behavior in the outer region and thereby avoids the common k-ω problem of over-sensitivity to inlet free-stream turbulence. The inlet turbulence intensity is 1.5% in this study. A range of this parameter between 1.5~2% has been suggested to be able to present the velocity information that is close to MRI data [36], and the same level of this parameter can be found in previous studies [18].

A finite volume solver (CFD-ACE+, ESI CFD) was employed for the numerical solution of the transport equations – the Reynolds averaged Naviér-Stokes equations. A second order accurate discretization (central differences) was used to solve the flow velocity; while an upwind scheme was applied to solve for the turbulence quantities, an approach which gives good overall accuracy with improved stability. Algebraic MultiGrid acceleration [37] was employed and the SIMPLEC-type pressure correction [38] was used for pressure–velocity coupling.

## Results and discussion

### Grid- and time step- independence

**Figure 3:**
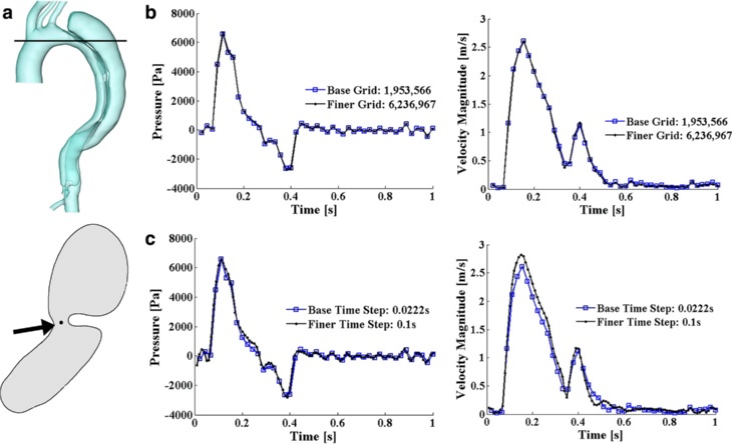
**Grid and time step sensitivity study. (a)** displays the studying point: the upper panel shows the cutting slice, and the lower one is the shape of that slice (viewing from the top) with an arrow pointing towards the studying point. **(b)** and **(c)** display the pressure and velocity magnitude variations at that point for grid independency study and for time step sensitivity study, respectively.

To confirm the insensitivity of the results to resolution, we conducted a grid independence analysis: apart from the base discretization, the solution on a finer grid with 6,236,967 cells has also been investigated. To compare the results of these two discretizations, a point near the proximal tear of the dissected aorta (a region of physiological interest and high numerical sensitivity), shown in Figure 3a, has been studied. Figure 3b displays the pressure and velocity magnitude variations at that specific point in the two models. The maximum discrepancies of the pressure and velocity magnitude during a heartbeat cycle between the two grids are 7.23% and 6.79%, respectively, and, for both, the exact same trends of the variable variations are observed. Therefore, for the purposes of our study the base resolution of 1,953,566 cells is deemed adequate.

Apart from the grid independency study, a test concerning the time step sensitivity of this unsteady simulation has also been conducted. Two temporal discretizations have been tested: 0.0222 s (45 steps per cycle) and 0.01 s (100 steps per cycle), both of which can present the pulse wave of the input velocity well. As the grid independency study, the pressure and velocity information of the same point has been studied. Figure 3c displays the parameter variations computed based on these two different time resolutions. The maximum discrepancies of the pressure and velocity magnitude during a heartbeat cycle are 9.57% and 7.42%, respectively, and very much the same patterns and trends have been observed, indicating the bigger time step (0.0222 s) is adequate for this unsteady simulation. The bulk of the results that we present below are, therefore, based on a grid with 1,953,566 cells and a time step of 0.0222 s.

### Flow patterns

The simulation has been carried out for 6 cardiac cycles to achieve a periodic solution. The results present below are based on information output in the final cycle. To satisfy the requirements of the k-ω SST turbulence model, a small y+ that is less than 2 should be achieved (where y+ is a properly non-dimensionalized wall distance). In this simulation, the maximum y+ during a cardiac cycle is 1.74, occurring at systolic peak, indicating the computational domain with 10 prismatic layers as the boundary layer is fine enough.

**Figure 4:**
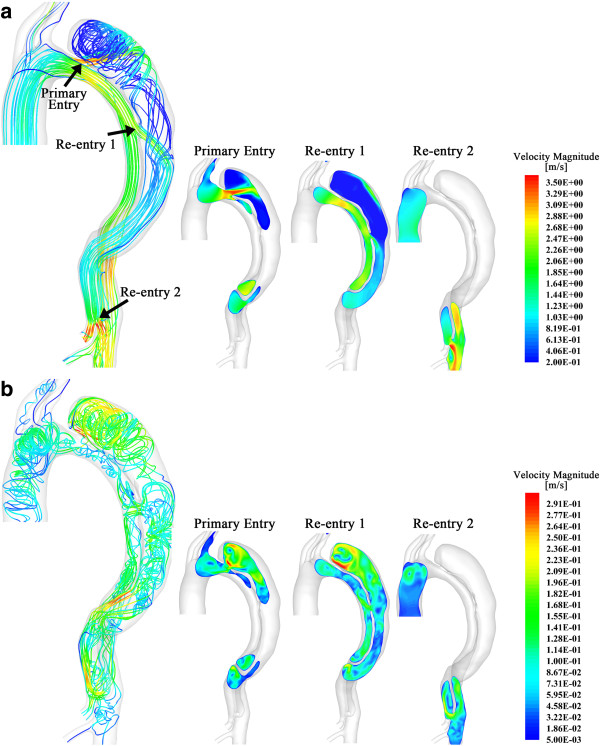
**Flow pattern within the dissected aorta.** The larger pictures on the left in **(a)** and **(b)** display the flow pattern at systolic peak and in the middle of diastole, respectively, by drawing lines that are tangential to the instantaneous velocity vectors. These lines are contoured by velocity magnitude. The smaller pictures on the right show the flow distribution by drawing the velocity magnitude contour at three slices, each of which crosses the entries that connect true and false lumen. Arrows in **(a)** indicates the positions and numbers of those entries.

Figure 4a and b show the flow pattern within in the dissected aorta at the systolic peak and in the mid-diastole, respectively. The larger picture on the left-hand side displays the lines that are tangential to the instantaneous velocity vectors, contoured by velocity magnitude. Since the flow at the regions close to the entries is of the most interest in dissected aorta studies, we also displayed slices showing velocity magnitude near the three entries in the three smaller pictures on the right. At the systolic peak (Figure 4a at 5.13 s), the flow is fairly organized within the entire true lumen; however, it presents highly helical features within the dissection. The flow is firstly accelerated when passing through primary entry. The small cross-sectional area of the primary entry increases the flow velocity and produces a jet in the false lumen. This jet flow is then obstructed and diverted by the boundary of the dissection (as shown in slice 1 of Figure 4a), spreads out over the dissection walls, and circulates as vortices in the upper region of the dissection with much lower velocity. Apart from the diversion at the primary entry, significant flow (specific fluxes will be discussed in the next section) from the true lumen also enters the false lumen through the re-entry 1 (numbers of the re-entries refer to the left panel of Figure 4a). As shown in slice 2 in Figure 4a, with the direction of the flow that is more parallel to the boundaries, the fast entering fluid makes the flow within the middle region of dissection more organized. In slice 3, due to the small cross-sectional area of re-entry 2, the flow re-enters the true lumen with very high velocity, which is up to 3.57 m/s, an important observation.

Figure 4b displays the flow pattern in the middle of diastole (at 5.76 s). The maximum velocity at this state is less than 1/12 of the systolic peak; as expected, the flow presents a markedly more vortical pattern. At this state, the flow in both the true and false lumens present highly helical features and the highest velocity (up to 0.3 m/s) occurs near the top of the dissection, when the flow circulates within the sac.

### Flow exchange between true and false lumen

**Figure 5:**
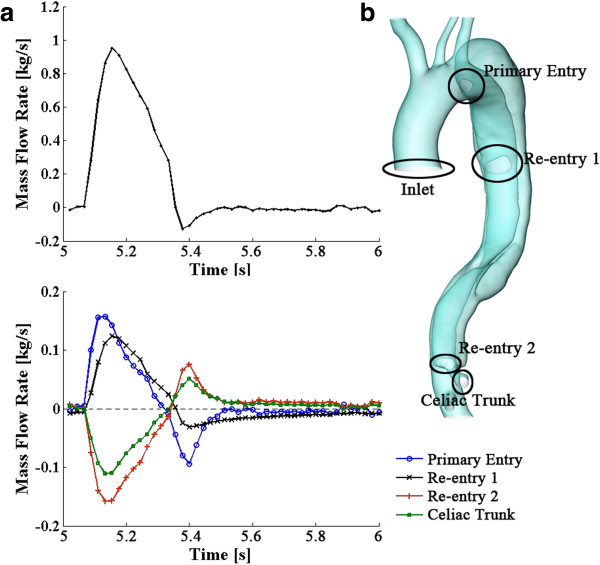
**Mass flow rate over a cardiac cycle (5~6 s).** The upper panel of **(a)** displays the mass flow rate at the inlet; while, the lower panel represents the variations of the mass flow rate at the three entries and the outlet of the celiac trunk; **(b)** represents the positions of the entries, the inlet of the aorta, and the outlet of the celiac trunk.

**Figure 6:**
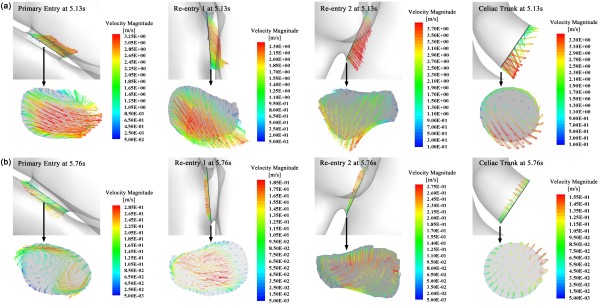
**Flow patterns at the entries and the outlet of the celiac trunk. (a)** displays the velocity vectors at these entries and outlet of the celiac trunk at the systolic peak; **(b)** displays the results at mid-diastole.

In order to calculate the communication between the aorta and dissection, the mass flow rate at the primary entry, re-entries and the outlet of the celiac trunk has been recorded, (Figure 5b). The upper panel of Figure 5a displays the mass inflow at the inlet; the lower panel represents the mass flow rates of the entries and the outlet of the celiac trunk. Figure 6 displays 2 snapshots of the flow at the entries and the outlet during systole and diastole, respectively.

During systole, about 28.11% of the total inflow injects into the dissection through the primary entry and re-entry 1, and then re-enters the true lumen through re-entry 2. During diastole, although the flow pattern becomes more complicated (as shown in Figures 4b and 6b), the flow exchange between the true and false lumen remains low; for instance, the maximum mass flow rate during diastole via the primary entry is about −7.39x10^-3^ kg/s, which is only 4.7% of the maximum flow rate at the primary entry during systole.

Over a cardiac cycle, transport through the primary entry, re-entry 1 and 2, and the outlet of celiac trunk is 7.55%, 6.59%, -8.79% and −5.35% of the overall flowing-in mass at the inlet (minus indicates outflow from the dissection). It should be noted that the mass flux over a cycle is a counteracting result of in- and out-flows towards the dissection. The absolute fluid mass that passes through those boundaries over a cycle is much higher. For instance, considering the time period between 5.04 s to 5.31 s, which covers most of the systole and during which the direction of the flow at each boundary remains the same, fluxes through the primary entry, re-entry 1 and 2, and the outlet of celiac trunk are 13.3%, 12.05%, -15.05% and −10.3% of the inflow at the inlet respectively, indicating a significant flow exchange between the true and false lumen. Moreover, the blood entering the inlet in a cardiac cycle can be obtained by calculating the area under the flux curve as shown in Figure 5a, which is 155.9 g. The fluid entering and passing the dissection can be computed by summing up the time-various positive (or negative) values of the fluid mass at each boundary of the dissection (positive indicates the flow enters the sac while negative indicates out-flows), and this is 61.8 g (39.65% of the inflow at the inlet). Indeed, as shown in Figures 5 and 6, the dissection not only diverts blood, it also largely disturbs the flow, inducing very high velocities near the entries and possibly turbulent flow patterns within the lumens, which reduces the available head for perfusion of the downstream organs.

### Pressure and wall shear stress distributions

**Figure 7:**
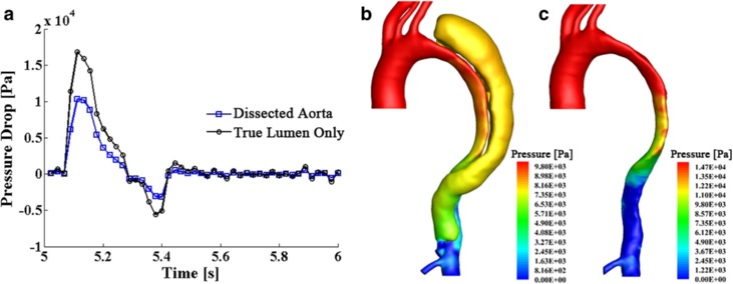
**Pressure drop curves over a cardiac cycle and the pressure distribution at systolic peak**. **(a)** displays the time-various pressure drop curves, which measure the pressure difference from the inlet at the ascending aorta to the end of the thoracic aorta; **(b)** and **(c)** respectively displays the pressure distribution at systolic peak for the dissected aorta and for the true lumen only.

The pressure drop from ascending to thoracic aorta is depicted in Figure 7a. The highest pressure drop occurs at systolic peak, when the blood flow is fastest. Figure 7b displays the pressure distribution at this state over the aorta and dissection walls. Compare to the pressure drop in normal thoracic aortic systems, which is less than 3,000 Pa (22.5 mmHg) [39], the pressure drop in this dissected aorta case is very high at systolic peak - 10,040 Pa (75 mmHg). In fact, hypertension is commonly found at the initial presentation of type-B aortic dissections [1,40]. With the dissected sac growing and continually compressing the aorta, it induces further true-lumen collapse, which further increases the blood pressure and may cause hypertensive crisis [41]. To confirm this, we artificially removed the dissection and studied the collapsed true lumen alone. Figure 7c represents the pressure distribution of this case with the pressure drop curve superimposed in Figure 7a. Even higher pressure drop is captured in this case. This indicates, as a buffering sac, the dissection contributes to diverting part of the flow (through a larger, albeit more complex cross-section) and thus relieving some pressure. On the other hand, the growth of the dissection and its extrusion towards the true lumen is the main factor to induce hypertension. This may imply, in stentgraft treatments, the implant should not only prevent the flow entering the false lumen, but more importantly, should be able to hold the true lumen open by displacing the intimal flap towards the false lumen (and overcoming the pressure load from within the false lumen), and therefore balance blood pressure. Indeed, a recent study of stentgraft placement in aortic dissections confirms that pressures can be greatly reduced by using a stentgraft to correct the shape of the collapsed true lumen [22].

**Figure 8:**
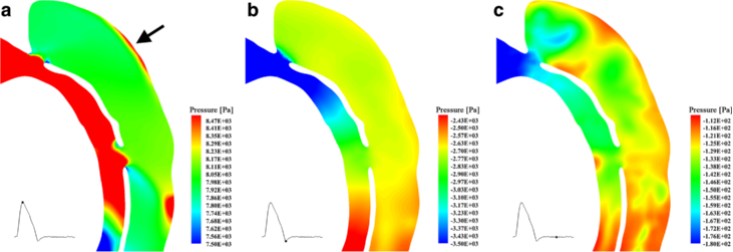
**Pressure distributions along a slice that crosses primary entry and re-entry 1.** The small image on the left bottom of each picture is the cardiac pulse wave, and the point on it indicates the time of the corresponding snapshots.

Pressure is a key factor of the further growth of dissection [42]. Figure 8 displays the pressure distribution along a slice that crosses the primary entry and re-entry 1, where most of the flow enters the false lumen. The results suggest that high pressure, up to 8,500 Pa, has been experienced by the outer wall of the dissection during systole, connected of course with jet impingement, as indicated by the arrows in Figure 8. Although the pressure along the outer wall of the dissection drops to a low level during diastole, in a long-time scale, this repeating mechanical force from the blood flow would cause the progressive enlargement of the false lumen and increase the risk of rupture.

**Figure 9:**
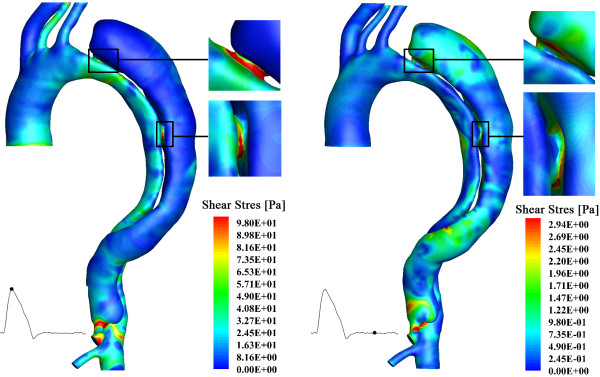
**Wall shear stress distribution of the aortic dissection system.** The left panel shows the results at systolic peak; and the right panel displays the results at mid-diastole.

Figure 9 represents the wall shear stress (WSS) distribution for the case studied. During systole, the dissected aorta system experiences very high WSS that is up to 89 Pa; while, during diastole, the WSS becomes much lower: the maximum WSS over this period is approximately 5 Pa. The highest WSS occurs near the entries, implicating the vulnerability of these positions to be further split; in fact, by decreasing the arterial shear stress, one can minimize the propagation of the dissection [43,44].

### Turbulence effects

**Figure 10:**
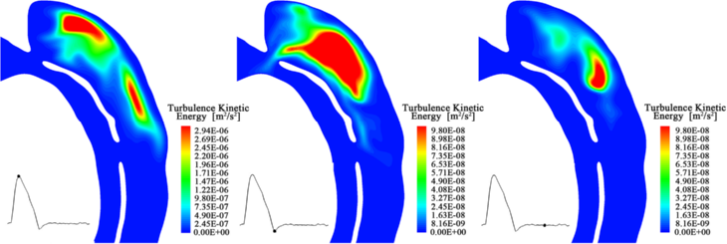
**Snapshots of turbulence kinetic energy distributions along a slice that crosses primary entry and re-entry 1.** The small image on the left bottom of each picture shows the corresponding time of the snapshots in a cardiac cycle.

Studies concerning turbulent features in blood vessels indicate that higher turbulence of the flow system would increase wall shear stress and lead to structural dilation of the walls [45,46]. In this study, turbulence kinetic energy (TKE) of the domain has been computed at each time step. It is the mean kinetic energy per unit mass associated with eddies at the turbulent lengthscale. For the particular geometry of this case, the highest TKE occurs in the upper region of the dissection, indicating higher velocity fluctuations exist in that region; connected of course with the sustained vortical motions observed in the mean velocities in those regions. Figure 10 shows three snapshots of the TKE distribution on a slice crosses the primary entry and the re-entry 1. Again, the maximum TKE occurs at systolic peak.

### Turbulence modeling vs. direct laminar computation

The high Reynolds number is occurred at systolic peak at the inlet of the model and near entries, indicating the turbulence patch emergence may be transient. To investigate the characteristics of the flow, and the potential of such modeling to become a clinical decision support tool, a comparison between turbulent and high resolution laminar solutions has been conducted. A very fine mesh with 28 million elements cells has been computed without a turbulence model and the results of this computation are juxtaposed to the turbulence models results presented above. It is interesting to note that the base grid and the much refined grid under laminar flow conditions yield results that are very similar. This possibly implies that the duration and extend of turbulence patch emergence may be relatively limited or, better put, may have limited effects on the global features of the flow.

**Figure 11:**
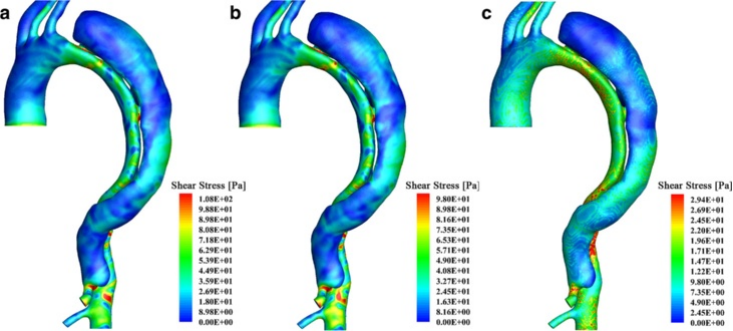
**Wall shear stress distribution of the aortic dissection system: a steady state test with systolic peak inflow. (a)** displays the result of the base mesh with a turbulence model; **(b)** is the result of the same mesh but based on a laminar model; while, **(c)** is the result based on laminar flow on a purely tetrahedral mesh without the prismatic boundary layer.

Moreover, the influence of the prismatic boundary layer elements has been studied. A simulation that involved purely tetrahedral elements (with approximately the same overall number of cells, 1,800,637 cells) is conducted under laminar flow conditions, and is compared to the results of the base grid under a turbulence model and under a laminar model, respectively. To expedite the comparison and computation times, these studies are based on steady simulations of turbulent and laminar flows at the systolic peak inlet velocity conditions. Variables at the point that has been used in the grid and time step independency studies (Figure 3a) have been investigated. Table 1 lists information regarding velocity magnitude, pressure, and shear stress at the monitoring point we have selected. Without the prismatic boundary layer, the laminar solution presents notable differences from the turbulent study and under-estimates the flow (the discrepancy is up to 30%); while, with near-wall mesh refinement, the laminar solution presents a much better correspondence to the k-ω SST model, especially for the results of velocity and pressure (the discrepancies are less than 1.3%). Figure 11 displays the wall shear stress distributions of the turbulent and laminar tests. The importance of near-wall resolution to capturing this parameter is clear. In this and all other comparisons (not shown here for brevity), the laminar study, with appropriate near wall resolution, captures the same trends of wall shear stress and other quantities as the turbulent model; while, the overall level of wall shear stress magnitude is lower. This result agrees with a report by Tan et al. [36] where, comparing turbulent and laminar models of the aortic system indeed showed that, when juxtaposed with MRI results, both laminar and turbulent simulations can correctly capture the overall flow patterns but the turbulence computations offer better quantitative agreement since magnitudes of the variables are slightly larger than those in the laminar model.

**Table 1:** Comparison of laminar and turbulent solutions

**Variable**	**Base grid turbulence model**	**Base grid laminar model**		**No boundary layer laminar model**	
		**Value**	**Discrepancy**	**Value**	**Discrepancy**
Velocity Magnitude [m/s]	3.02	3.01	0.33%	2.74	9.27%
Pressure [Pa]	5151.44	5087.07	1.25%	3628.83	29.57%
Shear Stress [Pa]	2.44	2.23	8.61%	1.85	24.18%

## Limitations

The results reported for one case may appear limiting. However, AD patients show highly individual features regarding the geometric information of the dissected aorta as well as the occurrence of intra-luminal tears, inducing difficulties to compare among various patients. The flow boundary conditions of this study were extracted from data of volunteers [27,39] and zero-pressure boundary conditions were assigned to the outlets of the model. The actual boundary information for the specific patient might present difference, which limits the accuracy of the simulated results. Since the current work focuses on the inter-luminal flow exchange and studies the loading distributions over the vessel wall, we believe the reported proportions of the fluid entering the false lumen and the weak points on the vessel wall, although not quantitatively precise, are meaningful in improving our understandings about the development of AD and are useful in assisting further interventional treatment design. More accurate flow analysis and validation of this case could be conducted when PC-MRI velocimetry and pressure measurements are available.

## Conclusions

We established a computational model of the dissected aorta by reconstructing the geometry from a CT dataset of a patient. Flow analysis indicates that (i) highest velocities occur close to the entries at systolic peak; and (ii) during systole, the true lumen experiences fairly organized flow, while vortical flow features dominate the false lumen; however, during diastole, disorganized flow patterns occur in both of the lumens.

The false lumen functions as an additional channel that diverts blood flow from the aorta. Along the flap that separates the two lumens there are normally several tears that allow flow to pass between the true and false lumen. It is very difficult to assess and measure flow entry and re-entry experimentally, however the computational model provides quantitative information about this communication between the two lumens (for this specific case) and reveals that, (i) 5.35% of the inflow at the inlet of ascending aorta is transported to the downstream arteries through the celiac trunk, which is perfused by the false lumen; and (ii) 39.65% of the overall inflow at the inlet enters and passes the false lumen during a cardiac cycle.

Loading distributions have also been investigated in this study. The aortic dissection system experiences very high pressures during the cardiac cycles. The outer wall of the dissection experiences a periodical thrust by the flow, which may induce further dilation. The wall shear stress level is also high in this case. The maximum wall shear stress is found near the entries, indicating the vulnerability of tearing at those positions.

Higher turbulence kinetic energy has been found in the upper region of the dissection. It suggests strong shearing and recirculation in those regions and has implications regarding platelet activation and thrombosis. Furthermore, laminar and turbulent solutions have been compared; due to the transitional characteristics of the flow, an appropriate fine near-wall boundary layer is required to achieve good simulation results. With proper resolution (and especially near wall resolution), the laminar solution presents very similar patterns as the turbulent one, although the magnitude of variables are smaller, which is consistent to the turbulent one, albeit small absolute magnitudes for the variables, as previous reports that compare the laminar and turbulent results with MRI data confirm [36].

In summary, such a patient-specific model of aortic dissection can provide detailed flow information of blood transport within the true and false lumen and quantify the loading distributions over the vessel walls. The analysis in this study contributes to understanding the hemodynamics of the dissected aorta system and the quantitative information provided can inform decision-making regarding stentgraft deployment.

## Abbreviations

AD: Aortic dissection; CT: Computed tomography; MRI: Magnetic resonance imaging; WSS: Wall shear stress; TKE: Turbulence kinetic energy.

## Competing interests

The authors declare that they have no financial or non-financial competing interests.

## Authors’ contributions

DC and YV performed the computational simulation and analysis of this work and drafted the manuscript. MME, HvTK, and DBöckler participated in the design of the study and performed radiological measurements. DBarber and RH developed the segmentation tool to reconstruct the patient-specific model. All authors read and approved the final manuscript.
